# Ultrasound-Guided Injection of a Tendon-Compatible Hyaluronic Acid Preparation for the Management of Partial Thickness Rotator Cuff Tear: A Case Report

**DOI:** 10.7759/cureus.20900

**Published:** 2022-01-03

**Authors:** Mariana Santiago, Filipa Gonçalves, Joana Martins, Tiago Lopes, José L Carvalho

**Affiliations:** 1 Serviço de Medicina Física e de Reabilitação, Centro Hospitalar Universitário de São João, Porto, PRT; 2 Serviço de Medicina Física e de Reabilitação, Centro de Reabilitação do Norte, Porto, PRT; 3 Serviço de Medicina Física e de Reabilitação, Centro Hospitalar e Universitário de Coimbra, Coimbra, PRT

**Keywords:** partial-thickness tear, treatment, shoulder pain, rotator cuff tear, ultrasound, injection, tendon compatible hyaluronic acid

## Abstract

Partial-thickness rotator cuff (RC) tear constitutes the most common cause of shoulder pain and disability. Its management is challenging, and a conservative approach is suggested as a first-line treatment. Nonetheless, minimally invasive approaches have been described in clinical trials, such as ultrasound (US)-guided tendon-compatible hyaluronic acid (HA) injection preparation in the rupture site.

HA is believed to fill the intradermal space and thus support the regeneration process by its integration in the damaged extracellular matrix. A reduced healing period required for a tendon tear when treated with a tendon-compatible HA preparation compared to placebo has been previously described in the literature, enabling a more rapid return to exercise.

The current study aims to provide a thorough analysis of a regular CrossFit practitioner case with a partial-thickness bursal-side RC tear of the anterior Supraspinatus (SS) fibers with 7 mm on the anteroposterior axis and 5 mm on the longitudinal axis in magnetic resonance imaging (MRI), that caused pain, and limited functional status. Two US-guided injections of a specific high molecular weight (one million Daltons) tendon-compatible HA preparation (12 mg/1.2 mL) separated by six weeks were performed. A supervised rehabilitation protocol was then followed and training was progressively introduced. In the 12 weeks follow-up visits, a reduction in pain intensity was noticed as well as an improvement of the functional status. At the six months, one year, and two years follow-ups, no pain and a normal joint function were observed, despite engaging in continuous overload and overhead activities during CrossFit practice. MRI was performed one year after the intervention presenting a reduction of the injury size and only a partial intrasubstance tear of 4 mm was observed in the SS tendon. US imaging in the two years follow-up presented an additional reduction in tear size to 3.9 mm length. No adverse effects were reported.

It is thus believed that US-guided injections of tendon-compatible HA on partial-thickness RC tears can be a feasible and effective treatment option in the management of this frequent pathology, and more studies, particularly randomized controlled trials, should be implemented to substantiate and validate this approach.

## Introduction

Partial-thickness rotator cuff (RC) tear constitutes the most common cause of shoulder pain and disability [1]. It occurs on the articular side of the tendon, in the bursal side, or in the intrasubstance [2].

Regarding the etiology of partial tears, bursal-side tears tend to be primarily caused by biomechanical subacromial impingement on a progressively degenerated tendon, while articular side tears result from trauma, repetitive movements, and/or an insidious start associated with degenerative age-related alterations on the tendon. In contrast, intrasubstance tears can result from internal tendon degeneration, namely cellular or extracellular matrix degenerative degradation [3].

A conservative approach is suggested as the first-line treatment of partial-thickness tendon tears, including topical, oral, or locally injected pharmacological approaches, rehabilitation treatment, and clinical exercise [1,4]. In the management of persistent symptoms after rehabilitation and topical and/or oral pharmacological treatments, steroid injections have been demonstrated to present a rapid pain relief impact but are temporary and are potentially able to damage tendon cells in repeated applications [5,6].

Hyaluronic acid (HA) is established in the literature for its intra-articular effects, as well as for stabilizing the synovial fluid chemistry and improving its functional quality [7]. Moreover, considering the tendon mechanobiology, some in vitro and animal in vivo studies have been revealing encouraging results in terms of the ability of HA to offer a similar positive impact on the extra-articular environment, not only by generating an improved tendon architectural organization but also by constraining the proinflammatory effect by restoring viscoelasticity and by stimulating the endogenous synthesis of HA [8]. These laboratory observations appear to support a reduced healing period required for a tendon tear when treated with HA rather than placebo, as described in the previous studies [1,8].

Based on the above information, HA is believed to fill the intradermal space and support thus the regeneration process by its integration in the damaged extracellular matrix [9].

The therapeutic efficacy of tendon-compatible HA injections under ultrasound (US) control directly to the region of the injured soft tissue has been documented in clinical trials [7,9,10], specifically of a specific high molecular weight (one million Daltons) tendon-compatible HA preparation (12 mg/1.2 mL), which was designed for optimal interaction with soft tissue due to its purity and biocompatibility profile. Histologically, this preparation is considered to promote healing without scar tissue [7].

## Case presentation

Pre-intervention consultation

A 36-year-old man, with a height of 1.72 m and a weight of 75 kg, working engineer and regular CrossFit practitioner, was presented in a specialized Intervention Physical Medicine and Rehabilitation (PMR) Consultation of a tertiary hospital with an insidious onset of mixed pain (mechanical and inflammatory) in the right shoulder, especially during repetitive overhead activities and when lifting heavy loads. There was no history of prior injuries to the affected extremity, namely previous trauma, as well as of personal or family comorbidities, or medication intake. He reported having functional difficulties in performing daily and sports activities, due to aggravating pain, and inability to sleep on the side of the affected shoulder. During the evaluation, he reported a seven out of 10 pain on the descriptive visual numeric scale (VNS) and scored 11 out of 35 on the University of California at Los Angeles (UCLA) Shoulder scale. Upon physical examination, the patient presented the shoulders in a slightly forward position, while he completed a complete articular range of motions in all planes and presented normal muscle strength. Specific tests were also performed for RC weakness, such as Patte, modified Patte, Lift-off, Belly press, and Jobe tests that were all negative. However, he presented a painful Jobe test and positive impingement tests (Yocum and Hawkins tests). In addition, superior or type three scapular dyskinesia (according to Kibler classification) was observed with excessive activation of the superior trapezius muscle [11].

**Figure 1:**
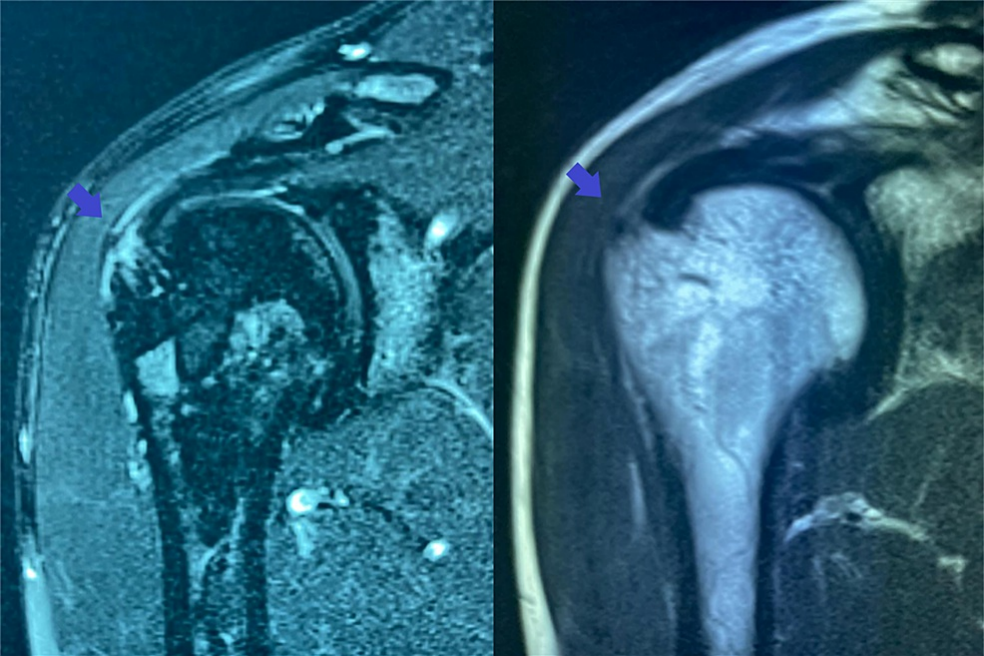
T2-weighted fat-saturated (left image) and T2-weighted (right image) right shoulder MRI presenting features of tendinosis of the SS and IS tendons with a partial-thickness bursal-side RC tear depending on the anterior SS fibers with 7 mm on the anteroposterior axis and 5 mm on the longitudinal axis. MRI: magnetic resonance imaging; IS: infraspinatus; RC: rotator cuff; SS: supraspinatus

The patient underwent a magnetic resonance imaging (MRI) two months before consultation, which revealed features of tendinosis of the supraspinatus (SS) and infraspinatus (IS) tendons with a partial-thickness bursal-side RC tear depending on the anterior SS fibers with 7 mm on the anteroposterior axis and 5 mm on the longitudinal axis (Figure 1). This RC injury could most likely result from chronic shoulder impingement syndrome [11]. The patient was thus suggested to proceed to an intervention.

Intervention

**Figure 2:**
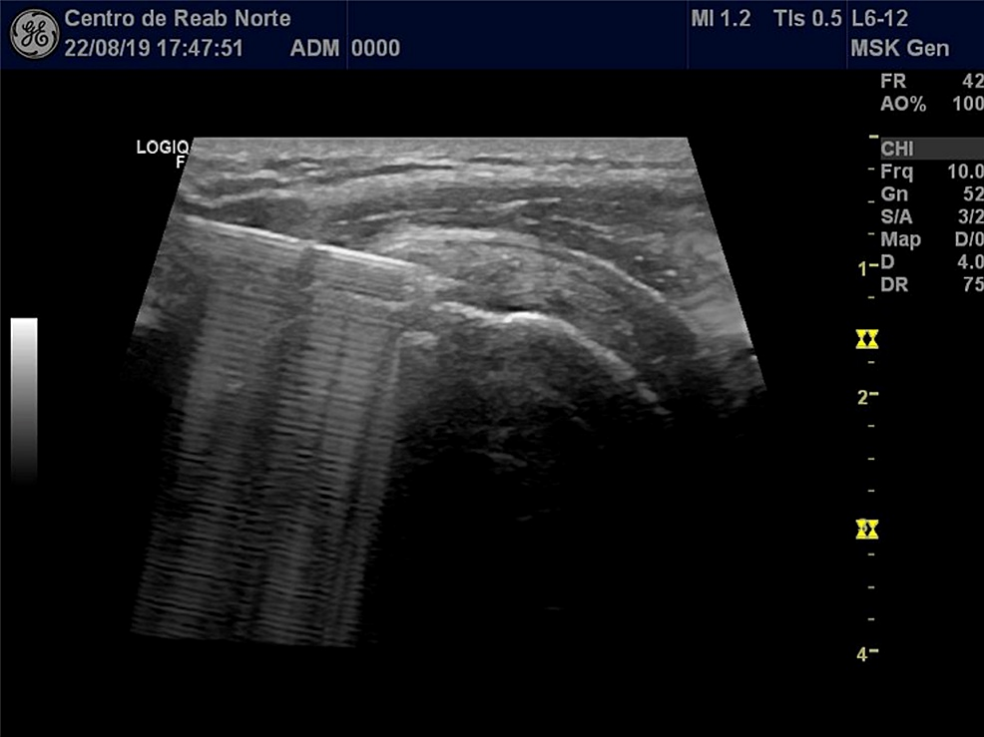
Visualization of the intervention: real-time in-plane intrasubstance injection of a tendon-compatible HA preparation (12 mg/1.2 mL) with a standard anterolateral approach under US guidance directly in the rupture site with a 22G/40 mm needle. HA: hyaluronic acid; US: ultrasound; 22G: 22 Gauge

The patient underwent two US-guided injections of a specific high molecular weight (one million Daltons) tendon-compatible HA preparation (12 mg/1.2 mL) at baseline and at the six weeks follow-up visit. The injections were performed in consultation by a PMR doctor with more than seven years of experience in Intervention PMR and applied a standard anterolateral approach under real-time US guidance, in the modified Crass position, directly in the target level (rupture site) with a 22-Gauge needle with 40 mm length needle, without applying a local anesthetic (Figure 2).

The procedure was well-tolerated, and no systemic or local adverse effects occurred after the injections.

The patient was requested not to take any non-steroidal anti-inflammatory drugs, corticosteroids (oral or injectable), or analgesics after the intervention, as well as to maintain the shoulder at rest after the HA injection for seven days without weight-bearing activities. A supervised rehabilitation protocol was then adopted with emphasis on periscapular and RC muscle strengthening. The intervention aimed at reducing posterior capsule and pectoralis minor restriction as well as restoring periscapular muscle balance through exercises promoting early and increased serratus anterior, lower, and middle trapezius activation while minimizing upper trapezius activity [12]. The training was subsequently progressively introduced and the patient was informed to perform pain-free exercises and to avoid increasing the number of repetitions in case of pain [1].

Post-intervention follow-up

Follow-up consisted of an algo-functional score 12 weeks after the procedure, in which the patient reported a two out of 10 pain on VNS and scored 35 out of 35 on the UCLA Shoulder scale. In the six months follow-up visit, the patient reported a 0 out of 10 pain on the VNS, maintaining the previous functional improvement scored by the UCLA Shoulder scale. At one and two years follow-up visits, the patient has not reported any pain (VNS 0/10) and he has a normal joint function, despite engaging in continuous overload and overhead activities during CrossFit practice. No adverse effects were reported during these two years of the follow-up.

**Figure 3:**
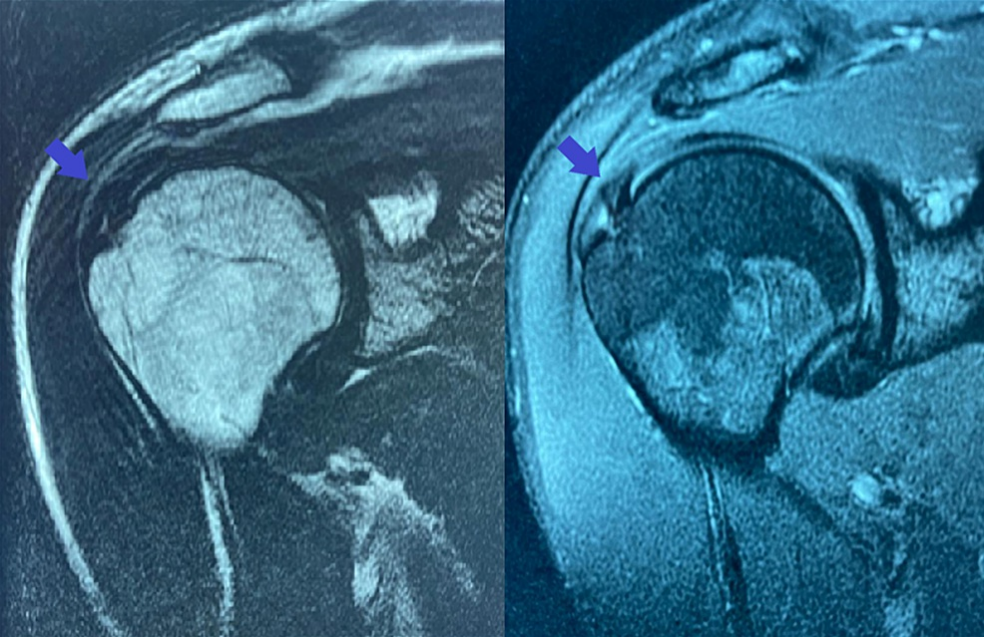
T2-weighted (left image) and fat-saturated proton density-weighted (right image) right shoulder MRI one year after the intervention, presenting reduction of the injury size and only a partial intrasubstance tear in the SS tendon of 4 mm length. MRI: magnetic resonance imaging; SS: supraspinatus

MRI was repeated one year after the intervention, presenting a reduction of the injury size and a partial intrasubstance tear in the SS tendon with 4 mm length (Figure 3).

**Figure 4:**
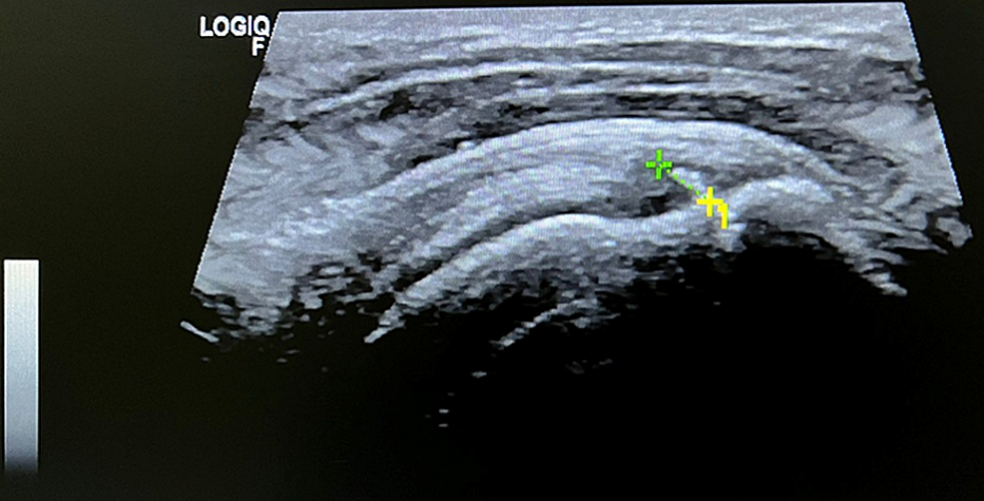
Ultrasound imaging (longitudinal view), performed by the PMR doctor in the two years follow-up visit presenting an additional reduction of the partial intrasubstance tear of the SS tendon with approximately 3.9 mm length. It is not clear whether the intrasubstance tear (marked location) corresponds to an actual fissure or an enthesopathic calcification (in this last case considering a complete regeneration of the rupture). PMR: Physical Medicine and Rehabilitation; SS: supraspinatus

In the two years follow-up, US imaging was performed by the same PMR doctor who performed the two injections, revealing a reduced partial intrasubstance SS tendon tear with approximately 3.9 mm length (Figure 4).

## Discussion

US-guided injections of tendon-compatible HA are an effective therapeutic option in the management of SS tendon tears, as it can reduce pain and improve shoulder function at short-term (two weeks) and medium-term (12 weeks) intervals [1].

This presented case was examined in order to provide new insights regarding the management of partial RC tears. A highly purified tendon-compatible HA, combined with an individualized rehabilitation program and gradual return to exercise, was effective in reducing pain and in improving shoulder function in short and medium-term intervals, but also in a long-term follow-up.

Based on the information provided by Cipolletta et al., the Delphi-based consensus was also followed in the current study that highly recommends the use of US guidance in procedures around the shoulder to provide imaging evidence of HA appropriate placement on the target level [1,13]. Although in that study a lack of a correlation between US and MRI was observed, in the present study both imaging modalities were considered, overcoming the anisotropy-dependent artifacts of US in the diagnosis of tendon tears [1].

Nevertheless, the current study highlighted the importance of US examinations on follow-up visits. Document progression, identification of changes of the tendon tear, as well as detection of new findings were considered crucial, as an enthesopathic calcification at the site of the previous rupture was revealed (Figure 4), corroborating the physiological role of HA in the homeostasis of tendons and its implications for regenerative medicine [14].

Moreover, a high molecular weight HA was used, which is considered to present an improved effect on tendon tears [14].

Laboratory analysis revealed reduced healing time with tendon-compatible HA rather than placebo, enabling a more rapid return to exercise, as was observed in the presented case [1].

## Conclusions

In this case report, the US-guided injections of a tendon-compatible HA, specific to manage soft tissue injuries, were correlated with pain resolution, enhanced structural and functional status, and a rapid return to exercise in partial-thickness RC tears. These enhancements were observed in the short-term, but also and most importantly they were maintained in the long-term follow-up.

It is that believed that US-guided injections of tendon-compatible HA on partial-thickness RC tears can be a feasible and effective treatment option in the management of this frequent pathology. Randomized controlled trials should be implemented to substantiate and validate this approach.

## References

[1] Cipolletta E, Mirza RM, Di Matteo A, Di Carlo M, Grassi W, Filippucci E (2021). Clinical efficacy of ultrasound-guided hyaluronic acid injections in patients with supraspinatus tendon tear. Clin Exp Rheumatol.

[2] Fukuda H (2000). Partial-thickness rotator cuff tears: a modern view on Codman’s classic. J Shoulder Elbow Surg.

[3] Carvalho CD, Cohen C, Belangero PS (2015). Partial rotator cuff injury in athletes: bursal or articular?. Rev Bras Ortop.

[4] Ryösä A, Laimi K, Äärimaa V, Lehtimäki K, Kukkonen J, Saltychev M (2017). Surgery or conservative treatment for rotator cuff tear: a meta-analysis. Disabil Rehabil.

[5] Coombes BK, Bisset L, Vicenzino B (2010). Efficacy and safety of corticosteroid injections and other injections for management of tendinopathy: a systematic review of randomised controlled trials. Lancet.

[6] Akpinar S, Hersekli MA, Demirors H, Tandogan RN, Kayaselcuk F (2002). Effects of methylprednisolone and betamethasone injections on the rotator cuff: an experimental study in rats. Adv Ther.

[7] Řezaninová J, Hrazdira L, Moc Králová D, Svoboda Z, Benaroya A (2018). Advanced conservative treatment of complete acute rupture of the lateral ankle ligaments: verifying by stabilometry. Foot Ankle Surg.

[8] Kaux JF, Samson A, Crielaard JM (2015). Hyaluronic acid and tendon lesions. Muscles Ligaments Tendons J.

[9] Merolla G, Bianchi P, Porcellini G (2013). Ultrasound-guided subacromial injections of sodium hyaluronate for the management of rotator cuff tendinopathy: a prospective comparative study with rehabilitation therapy. Musculoskelet Surg.

[10] Tomaszewski W (2015). Is the use of STABHA™ for supplementation of damaged extracellular matrix of soft tissues in the musculoskeletal system an effective treatment of acute injuries and tendinopathies? (Article in Polish). Ortop Traumatol Rehabil.

[11] Kibler WB, Uhl TL, Maddux JW, Brooks PV, Zeller B, McMullen J (2002). Qualitative clinical evaluation of scapular dysfunction: a reliability study. J Shoulder Elbow Surg.

[12] Cools AM, Dewitte V, Lanszweert F (2007). Rehabilitation of scapular muscle balance: which exercises to prescribe?. Am J Sports Med.

[13] Sconfienza LM, Adriaensen M, Albano D (2020). Clinical indications for image-guided interventional procedures in the musculoskeletal system: a Delphi-based consensus paper from the European Society of Musculoskeletal Radiology (ESSR)-Part II, elbow and wrist. Eur Radiol.

[14] Osti L, Berardocco M, di Giacomo V, Di Bernardo G, Oliva F, Berardi AC (2015). Hyaluronic acid increases tendon derived cell viability and collagen type I expression in vitro: comparative study of four different Hyaluronic acid preparations by molecular weight. BMC Musculoskelet Disord.

